# Barriers to Effective Postmenopausal Osteoporosis Treatment: A Qualitative Study of Patients’ and Practitioners’ Views

**DOI:** 10.1371/journal.pone.0158365

**Published:** 2016-06-29

**Authors:** Sophie Alami, Lucile Hervouet, Serge Poiraudeau, Karine Briot, Christian Roux

**Affiliations:** 1 Interlis, Paris, France; 2 Centre de Sociologie des Organisations, Institut des Sciences Politiques, UMR 7116, CNRS, Paris, France; 3 U1153 Institut National de la Santé et de la Recherche Médicale; PRES Sorbonne Paris Cité, Université Paris Descartes; Service de Rééducation et de réadaptation de l’appareil locomoteur et des pathologies du rachis, Hôpital Cochin, Assistance Publique–Hôpitaux de Paris, Paris, France; 4 U1153 Institut National de la Santé et de la Recherche Médicale; PRES Sorbonne Paris Cité, Université Paris Descartes; Service de Rhumatologie B, Hôpital Cochin, Assistance Publique–Hôpitaux de Paris, Paris, France; Klinikum rechts der Isar—Technical University Munich—TUM, GERMANY

## Abstract

**Background:**

Only a minority of patients at high risk for osteoporotic fracture receive treatment.

**Objective:**

Study patients’ and physicians’ views regarding postmenopausal osteoporosis (PMO) to identify impediments to good care.

**Methods:**

A qualitative study involving 18 physicians and 37 women (age 57–87) with PMO.

**Results:**

All women interviewed considered PMO to be somewhat normal wear-and-tear associated with old age. The women identified a large number of "causes" for osteoporosis but finally viewed it as chance. They all described its progression as slow. Three representations of PMO severity were identified: some women tended to interpreted it as benign (21), others tended to dramatize it (11), and the rest were uncertain (5). These representations did not appear linked to age or fracture. Even the women who associated fracture and PMO were uncertain of the link between them. Fractures were considered to be random events, independent of osteoporosis. Women received general life-style recommendations from their physicians positively, but did not connect them specifically to osteoporosis. Indeed, these recommendations, along with the fear of side effects, the absence of tangible results of treatments, the view of PMO as a natural process, and the representations of PMO severity are factors that may deter treatments and impact compliance. As for the physicians, they identified eight risk factors, recognizing menopause as central to PMO and recognized the link between risk of fracture and PMO. However, some considered the impact of fractures to be limited in time, and viewed PMO as a "benign" disease. Seeing the progression of PMO as slow and inevitable reduced their urgency to diagnose and treat it as compared to other diseases. Some physicians acknowledged limited mastery of the existing therapeutic arsenal and unsuccessful handling of patient compliance.

**Conclusion:**

Women’s and physicians’ perspectives on PMO converged to trivialize postmenopausal osteoporosis and thus disqualify it as a legitimate disease. A better understanding of women’s and physicians’ views, practices, and concerns related to PMO can improve osteoporosis management.

## Introduction

Only a minority of patients at high risk of a fragility fracture receive treatment [1, 2, 3], despite the availability of guidelines for appropriate care [4] and, the rates of treatment, even after hip fractures, have fallen in recent years [5–9]. Prospective studies have shown that most osteoporotic fractures are associated with increased morbidity and substantial increased risk of repeated fractures [10]. Attention has been paid to the increased risk of mortality after hip fractures [11, 12], vertebral fractures [13], and other non-vertebral non-hip fractures [12], with a particular impact of refracture [14]. Subsequent fractures cluster in time after the first fracture [15, 16], offering an optimal therapeutic window. In prospective placebo-controlled studies with appropriate methodology, available medications have been shown to decrease the risk of fractures in post-menopausal women with previous vertebral [17] or hip [18] fracture, and/or low bone mineral density [19, 20]. However, even after a fragility fracture, patients do not associate their fracture with osteoporosis or appreciate the actual risk of complications [21, 22]. Moreover, concerns about adverse effects of osteoporotic medications [23] recently received considerable public exposure [24], and adverse news media may have diminished prescriptions [25].

Positive outcomes in osteoporosis hinge on relevant medical prescriptions and appropriate use of medications by patients. Thus, the aim of our study was to identify social representations of postmenopausal osteoporosis (PMO)—i.e. the beliefs, norms, opinions, and perceptions related to the illness that shape how individuals interpret and respond to their experiences—and their effects on efficient management of osteoporosis and fracture prevention. The present study used qualitative methodology to explore the patients’ and practitioners’ views regarding PMO and to identify potential improvements in medical care strategies. Our research questions were: (i) What are women’s and practitioners’ views of the disease and its treatment? (ii) What are their expectations in terms of medical care? (iii) Are there any differences between the two groups’ views and priorities? (iv) To what extent do women’s and practitioners’ views influence treatment breaks and refusals?

## Methods

### Design

To address the goals of this study, a qualitative research strategy was developed to deal with exploratory, comprehensive, and descriptive concerns [26]. An inductive enquiry consistent with a grounded-theory approach [27] was used in accordance with qualitative guidelines [28–30]. Face-to-face semi-structured interviews were conducted with physicians and women with PMO. A focus group was also organized to further probe the data collected in individual interviews: projective and associative techniques were used to discuss potential issues related to PMO and its treatment [31].

### Participants and sampling

The study included 37 women with PMO and 18 physicians. To be eligible for the study, the women had to have been diagnosed with PMO by a bone density test and to have received a long-term prescription for an anti-osteoporotic treatment. The guiding principle of our sampling procedure was to favor diversity. Women were purposively sampled to ensure this diversity according to age (21 participants between 50 and 69 and 16 aged 70 or over), symptoms (16 fractures, 7 hip fractures), and self-reported compliance (19 women declaring to be adherent) [S1 Table]. A focus group of 4 women without fracture, age 50 to 69, who declared medical compliance, was formed to gain a deeper understanding of why some younger asymptomatic interviewees were compliant even though their condition might seem to impede medical adherence. Individuals with other forms of osteoporosis were excluded in order to clearly circumscribe the results to PMO. The physician sample included 18 persons with public, private, and mixed practices, from the 3 main medical specialties (8 general practitioners [GP], 5 gynecologists [G] and 5 rheumatologists [R]) involved in the management of osteoporosis in France. All the physicians interviewed confirmed that they had women suffering from PMO in their patient base.

### Recruitment

Multiple recruitment strategies were used to maximize the diversity of participants. We used the patient lists of 2 doctors of the research team (CR, KB) to recruit 15 women and 3 physicians. The other participants (22 women and 15 physicians) were recruited through various networks: those of the first participants, of the 2 research team sociologists [SA, LH]) of 1 private nurse, and of 2 professional recruiters in 2 distinct regions, using flyers and direct contact. Interested women either contacted the sociologists in charge of the study or agreeded to be phoned with further information to set an appointment for an interview. Written informed consent was obtained from all the women who participated in the study. All physicians gave oral informed consent.

### Ethics approval

The study protocol was approved by the ethics committee Comité de Protection des Personnes Ile de France III of Tarnier–Cochin Hospital, Paris. All patients received written information presenting the aims, methodology, funding, and institutional affiliation of the researchers, as well as the potential risks and benefits of the study. They gave written informed consent to participate in the study. The study also conformed to the regulations concerning the use of private personal data, and was approved by the French agencies that certify research and enforce the Freedom of Information Act, namely the Comité Consultatif sur le Traitement de l’Information en matière de Recherche dans le domaine de la Santé and the Commission Nationale de l’Informatique et des Libertés. The research was conducted according to the Declaration of Helsinki principles.

### Interview and focus group protocols

The interviews followed semi-structured guides with opportunity for open-ended discussion. As analysis progressed, interviewers could thus pursue new topics when they arose, elicit specific information, and remain open to unexpected topics in the follow-up questions. They could also review the interviews conducted and adjust their guides.

The patient and physician protocols covered the same topics, allowing viewpoint crossing and data comparison for the issues discussed. The protocol for women with PMO comprised: general views, personal experiences, attitudes towards treatment and decision-making processes, outcomes, and expectations. It combined a thematic structure (views of PMO, its impact in daily life and subsequent adjustments, therapeutic journey, treatment, and expectations) with chronological sequences to examine in detail the therapeutic journey as well as the patient-physician relationship. The physician protocol covered doctors' views on osteoporosis, its management (from diagnosis to therapeutic decision-making processes), patient-physician interactions, and physicians’ expectations. The protocols included representations, reported practices related to PMO, and expectations.

### Procedure

The women were interviewed between March and July 2014. The interviews lasted 1 hour 40 minutes on average (ranging from 1 hour to 2 hours 51 minutes). The interviews took place in participants’ homes (25) or at sociologists’ offices, depending on the women’s preferences. The focus group lasted 3 hours and was conducted at Cochin Hospital (Paris, France) in May 2014.

The physician interviews were conducted between December 2013 and March 2014. They took place in the doctors’ offices (15), hospitals (1), or homes (2) and lasted 1 hour 5 minutes on average (ranging from 1 hour to 1 hour 30 minutes).

The interviewers ensured that all aspects of the study were sufficiently explained during the interviews and before the focus group. The interviews and the focus group were audio-recorded with permission. They were transcribed verbatim and the audio recordings were erased within 15 days. All interviews were conducted with a commitment to respecting the anonymity of respondents.

### Data analysis

The sociologists used inductive thematic analysis to identify and analyze themes and subthemes in the data. They read a preliminary series of interviews and developed a preliminary list of coding categories. As the fieldwork and the analysis progressed, they refined the list (by expanding, detailing, splicing, or linking codes) and modified it as new insights about the data were found. The sociologists discussed the coding frame, fine-tuning the bases of the coding categories. This list provided a content analysis grid to code the entire data. Sessions of peer debriefing with osteoporosis specialists (KB, CR, SP) were held to ensure a high level of inter-researcher consistency.

## Results

### Sample characteristics

The women with PMO came from 6 different regions of the country. Tables 1, 2, 3 and S2 Table show the diversity of the socio-demographic and medical characteristics of the sample. Fifteen out of 37 were treated by 2 different doctors in the same hospital department; the others consulted all different physicians.

**Table 1:** Women’s demographics (n = 37).

Socio-demographic characteristics		N
**Age group**		
	50–59	4
	60–64	13
	65–69	4
	70–75	6
	>75	10
**Median age (range)**	67 (55–87)	
**Living situation**		
	Living alone (single/widow/divorced)	17 (3/6/8)
	Living with others (married/cohabiting)	20 (19/1)
**Employment status**		
	Employed	6
	Not employed	3
	Retired	28
**Education**		
	No diploma	4
	≤ high school diploma	13
	University degree	12
	Undeclared	8
**Living area**		
	Rural	4
	Semi-rural	6
	Urban	27

**Table 2:** Women’s clinical characteristics (n = 37).

Declared clinical characteristics			N
**Fracture**			
	**With fracture**		16
	- Age 50–69		8 (5 multi-fractured)
	- Age ≥ 70		8 (5 multi-fractured)
**Sites of fracture**			
	**Single fracture**		6
	- Hip		3
	- Wrist		1
	- Vertebra		2
	**Multi-fractured**		10
	- Hip + other sites (foot, elbow, vertebra, hip, humerus, ankle)		4
	- Other sites (wrist, elbow, rib, malleolus, humerus, ankle, kneecap, coccyx, toes)		6
**Other diseases declared**			25
	**Primary risk factors**		2
	- Anorexia (prior to osteoporosis)		
	**Secondary risk factors**		
	- Asthma		3
	- Cancer		5
		- Uterus	1
		- Breast	4
	**Unrelated to osteoporosis**		
	- osteoarthrosis		17
	- Hypercholesterolemia		4
	- Hypertension		1
	- Chronic obstructive pulmonary disease		1
	- Stroke		2
	- Scoliosis		1
	- Gammopathy + Meniere’s disease + heart problems		1
	- Hypothyroidism		1

**Table 3:** PMO Treatments prescribed to women at interview time.

Last PO treatment prescribed (other than vitamin D and calcium supplementation)	Number of women	Compliant behavior	Noncompliant behavior (Refusal or premature treatment breaks)
Biphosphonates	23	12	11
Denosumab	5	2	3
Raloxifène	4	3	1
Strontium ranelate	3	1	2
Tériparatide	1	1	_
Hormonal replacement therapy	1	_	1
Total	37	19	18

The physician sample included 18 persons of both genders (10 women, 8 men), with a diversity of age (4 under 45, 9 between 45 and 60, and 5 over 60), of geographic location (from 5 different regions, 1 practicing in a rural environment and 3 in a semi-rural setting), and of practice settings (1 working solely in a public hospital; 11 with private practice; 6 with mixed activity) [Table 4].

**Table 4:** Physicians’ characteristics.

Physician number	Medical specialty	Sex	Age	Localization	Practice setting
GP1	General practitioner	Woman	44	Semi-rural	Private
GP2	General practitioner	Woman	31	Urban	Private
GP3	General practitioner	Woman	47	Semi-rural	Private
GP4	General practitioner	Woman	52	Urban	Private
GP5	General practitioner	Men	59	Rural	Private
GP6	General practitioner	Men	76	Urban	Private
GP7	General practitioner	Men	53	Urban	Private
GP8	General practitioner	Men	48	Semi-rural	Private
RH1	Rheumatologist	Woman	34	Urban	Mixed activity
RH2	Rheumatologist	Woman	53	Urban	Mixed activity
RH3	Rheumatologist	Men	34	Urban	Public hospital
RH4	Rheumatologist	Men	45	Urban	Mixed activity
RH5	Rheumatologist	Men	67	Urban	Mixed activity
GY1	Gynecologist	Woman	63	Urban	Private
GY2	Gynecologist	Woman	59	Urban	Mixed activity
GY3	Gynecologist	Woman	59	Urban	Private
GY4	Gynecologist	Woman	61	Urban	Private
GY5	Gynecologist	Men	62	Urban	Private

### The women’s views of postmenopausal osteoporosis

#### Bone and osteoporosis

Osteoporosis is a reality that the women seemed familiar with, but distantly so. Information on osteoporosis gleaned in the media or from family and friends did not necessarily make them feel concerned. Women who felt that they were youthful and had a healthy lifestyle, who were professionally active, and who fulfilled family duties felt little concern: *"(Had you heard of osteoporosis before the density test?) Yes*, *of course*, *but I was oblivious*. *I felt young*." (W18).

All the women interviewed clearly associated osteoporosis with bone disorder but had little to add. Bones were invisible and unnoticeable in everyday life: "*I feel my joints*, *but not my bones*."(W36). They did not all perceive their bones as requiring care. Bone deterioration was not viewed as life-threatening but merely as jeopardizing posture and mobility. When bone preservation practices were mentioned, they concerned diet (dairy consumption) and physical activity ("moving"), but were actually part of preserving overall health.

Osteoporosis was not always perceived as a disease, but rather as natural bone deterioration, an inevitable part of aging. The women described osteoporosis as bone damage, "decalcification", or simple "fragility". Three metaphors described the action of osteoporosis on bone: erosion ("*a tree losing its bark*"), nibbling away ("*lace*") and softening ("*marshmallows*").

#### Causes of osteoporosis

When discussing causes, all women were uncertain about the reasons for their own osteoporosis: "*I don’t have any hypotheses; I think it's a gamble*!" (W19). When further queried, they cited 11 possible "causes": aging (mentioned by 26 out of the 37 women surveyed), menopause (29 mentions), female gender (3), heredity (13), poor lifestyle (20), calcium failing to attach in bones (4), psychological frailty (2), pregnancy (2), diseases (6), iatrogenic effects of drugs (4) and "*chance*" (4). Menopause was cited as a cause of osteoporosis, but the women did not spontaneously describe their osteoporosis as postmenopausal osteoporosis. The women felt that these various causes did not automatically provoke osteoporosis in everyone or lead systematically to the same manifestations or severity.

#### Symptoms and severity of osteoporosis

Two categories of symptoms were identified by the women interviewed: those directly associated with osteoporosis (fractures, loss of height, and curvature of the spine) and those associated with fractures (pain, fatigue, loss of mobility). However, they observed, the symptoms were invisible or indistinguishable from the signs of old age or other diseases [Table 5]. In a free association exercise, the focus group participants compared osteoporosis to animals considered "sneaky," such as "snakes" and "cats", a disease that "*works quietly without saying anything*, *without hurting*." The "invisibility" enabled women to distance themselves from the experience of illness: "*Osteoporosis is a silent disease; I don’t have the impression that I’m ill*." (W6). The progression of osteoporosis was seen as "slow," imperceptible ("*you don’t notice anything*"), and uncertain ("*we'll see what happens*"), prompting W24 to compare it to "*chef's surprise*." Thus, they imagined three possible outcomes: the disease would stabilize, bone density would be restored, or irreversible damage would produce a therapeutic impasse.

**Table 5:** Women’s views concerning PMO symptoms.

Symptoms related to osteoporosis	Selected quotations
**Imperceptible symptoms:**	
	"It didn’t cause any pain. There was nothing that told me I had osteoporosis." (W20, age 72).–"(If you think of osteoporosis, what are the first words that come to mind?) Painless."–"You don’t feel it."–"You can’t see it." (Focus group, age 50 to 69).
**Indirect symptoms of osteoporosis associated with fractures:**	
- Fatigue	"Sometimes I’m so tired that I go to bed and I can sleep during the day, because of the pain (from osteoporosis)." (W11, age 64).
- Pain	"I don’t think [osteoporosis] is painful; it’s the consequences that are." (W8, age 66).
- Curved spine	"Because of the compression of the vertebrae, you hunch over more and more and you can’t straighten up." (W6, age 70).
**Symptoms viewed as nonspecific, indistinguishable from signs of aging or other illnesses:**	
- Pain	"I’m in pain; it hurts all over, all the time. Is it arthritis, is it osteoporosis? I don’t know." (W34, age 60).
- Fracture	"I think anybody can have a fracture. I wound up stepping into a 5-cm hole. I twisted my foot and hit it here on the asphalt… I think you could fracture something the same way without osteoporosis." (W12, age 68).–"If you fall, even if you don’t have fragile bones, you can break something, no? It depends on how you fall, that’s all." (W3, age 79).–"It seemed normal to break my femur neck after a certain age." (Focus group, age 50 to 69).
- Loss of height	"In any case, over time, there’s always a sort of compression, whether you like it or not.” (W3, age 79)–"I lost two centimetres. I was 1m65 tall; I’m not 1m65 anymore, but I think that’s normal. After a while, everybody loses a bit (of height)." (W34, age 60).

Four indicators defined the severity of osteoporosis for them: early onset of the disease, spontaneous fractures, test results (in particular the T-score), and their doctor’s remarks. They had two different reactions to osteoporosis: some relativized and dissociated the risk: "*It's not life-threatening*. *That's why people don’t pay so much attention to it*." (W36). Others talked about the risk of injury and disability: "*You’re afraid it’s going to happen again; you think*, *you fall and it starts all over again*. *It’s an endless circle*. *It took a fall to disable me like this*." (W26). In these testimonies, mortal risk was mentioned (W1, W8, W19 and 1 focus group member) but remained abstract; interviewees did not clearly perceive the conditions that led to death.

Although fracture was considered to be an indicator of severity, it was also described as a non-specific symptom [Table 5] that occurred randomly and independently from osteoporosis. Thus, uncertainty prevailed concerning the link between PMO and fracture: "*If you fall*, *even if you don’t have fragile bones*, *you can break something*, *right? It depends on the fall*!*" (W3)*. *"I think (fractures) can happen to everyone… I think you can have one all the same*, *without osteoporosis*." (W12).

The uncertainty about the relation between fracture and osteoporosis may have been conveyed by their physicians. One focus group member stated: "*A rheumatologist told me that certain women with osteoporosis may fall and not sustain fractures*. *So you don’t know what the link really is*." Uncertainty may also have resulted from a lack of information: "(*Did the doctor talk to you about spontaneous fracture?) No*, *no one ever mentioned it*." (W22). "(*Did your regular doctor or your gynecologist link osteoporosis and the risk of fractures?) No*, *no*, *they never talked about it*. *You know*, *there’s not really much time to stop and talk*." (W36).

Finally, the women’s representations of PMO severity were divided between: a "dramatized" view of osteoporosis as a disease with serious risks, particularly in terms of damage to self-image and dependence (11 women out of 37) and a view that "relativizes" PMO as a disease (21 women). A few (5 women) vacillated between the two opposites (Table 6). In this qualitative study, neither experience of fracture nor age or the naturalistic representation of PMO appeared to determine the differing views of the severity of PMO.

**Table 6:** Women’s views of PMO severity.

Views of PMO severity	N	Symptom
**"Relativization"**	21	9 fractured
**"Dramatization"**	11	5 Fractured
**Uncertain**	5	2 Fractured

#### Treatments of osteoporosis

Representations of PMO were not the only factor that conditioned women’s behavior and treatment practices. Women who were not compliant expressed either a dramatized, a relativized, or an uncertain representation of PMO: 4 of the 11 women who had a dramatized vision of osteoporosis were non-compliant, as were 10 of the 20 who relativized PMO as a disease and 4 out of 5 who felt uncertain. Indeed, comments on adherence involved not only representations of the disease but also those of treatments.

The women differentiated between 3 types of treatments: general health advice, supplements, and anti-osteoporotic medications. The general life-style recommendations that women received were physical exercise, dietary modifications, and to a lesser extent, strategies designed to prevent falls. Those recommendations were well received; however, because they were considered general rules for good health and aging, they were not seen to be essential to osteoporosis prevention. In addition, for some, exercise seemed too daunting or too difficult to perform in their condition. The recommendation to consume dairy products met with resistance (10 women); some considered them unhealthy, unappealing, or full of cholesterol.

Vitamin D and calcium supplements, on the other hand, were viewed as beneficial for bone and deemed "safe" *("it can’t do me any harm*." W13); "widespread" ("*we’ve always taken it"* W8); and "natural "("*for me*, *they aren’t medicine*. *I like them; I think there are fewer chemicals*." W8). Certain women had reservations concerning the tangibility of the benefits and reported side effects such as nausea, difficult digestion, and a persistent unpleasant taste in the mouth.

The anti-osteoporotic treatments generated more fear. These products were considered harmful (19 women, 17 having cited a biphosphonate as the last treatment they were prescribed), and the interviewees interpreted the side effects experienced or reported by relatives, doctors, or the media and the fact that treatments are limited in time as dangerous indicators: "*It must be somewhat stronger*. *Definitely*, *since some people say to stop after 6 years*. *Why would they say that if there were no effects? The doctor spoke of saturation and said that you had to take a break*. *According to him*, *it was really indispensable*" (W7). Treatments were described as burdensome and ineffective, and some interviewees found the risk-to-benefit ratio unfavorable (19 women, 17 non compliant and 2 compliant). The risk of fracture could be seen as uncertain; some women had fallen but never had a fracture. Furthermore, the interviewees pointed out that fracture could be avoided by being careful not to fall. It was also described as damage that surgery can repair nowadays. Even when fracture was seen as a possible risk, medication could still be perceived as something to avoid. "*I haven’t tried any of them*. *When I see the side effects*, *that’s it*. *I’d rather have a fracture than find myself with diseases*, *cancer*, *or something that may be more serious*. *It’s true that breaking a femur is no fun*, *but it may be less complicated in any case*!" (W16). The experienced or perceived negative outcomes associated with anti-osteoporosis treatments appeared to be more tangible than potential positive benefits, which were not directly noticeable. Moreover, taking anti-osteoporotic medications did not always seem to lead to an improvement in their bone density and did not systematically prevent fracture. The interviewees evaluated treatment efficacy based on their overall representations concerning PMO and on their expectations for the treatment. The stabilization of bone density, for instance, represented success for some and failure for others.

The women interviewed manifested clear expectations concerning their anti-osteoporosis treatment: it should be efficient, safe, easily administered and as inexpensive as possible. These expectations coincided with general expectations towards pharmaceuticals, echoing an overall mistrust of allopathic treatments and an interest in lifestyle advice and alternative therapies. The women also expressed a need for more information about both the disease and its treatment. They wanted information about the goals of their treatment, its duration, its implementation, its side effects, possible causes of failure, ways of increasing its effectiveness, alternatives to pharmaceutical therapy, and recent therapeutic advances. They expected information about the disease itself—progression, causes, symptoms, and prognosis: "*Explain to me what it is in more detail*. *Show me a bone and tell me what's wrong*, *we can fix it this way… They need to provide some education*. *This is something that happens to you*, *you don’t know anything about it; you're not ready for it*." (W13). The women thought that more public information would prevent osteoporosis and would foster awareness: "*There is no campaign telling people to get a bone density test*. *We aren’t told to think about our bone density or to think that a fracture of the femoral neck might cause problems*. *No one talks about it*." (W8).

### Physicians’ views of osteoporosis

The physicians’ comments on PMO presented common characteristics with those of patients even though they involved greater medical knowledge and awareness of cost. What they said about osteoporosis in general and PMO in particular—its risk factors, symptoms, severity, associated risks, causes, progression, and management—revealed representations that favor minimization or even disqualification of the disease and demonstrated the difficulty of dealing with PMO. Like the women interviewed, the physicians described osteoporosis as a "natural" process of human aging, leading some professionals (mainly GPs and gynecologists) to challenge the labelling of PMO as a disease: "*In my view*, *it’s not a disease*, *in that it’s the aging process*, *and we will all be osteoporotic*." (GP8).

The cause of PMO was described as an imbalance in bone remodelling resulting from hormonal disorders or vitamin-D deficiency. Eight risk factors were identified: menopause, early menopause, heredity, lifestyle (smoking, excess consumption of alcohol, physical inactivity, vitamin-D deficiency due to lack of sunlight exposure), diet (calcium deficiency and "women who have dieted all their lives"), body weight (underweight or overweight), treatment with corticosteroids, and diseases such as diabetes, hyperthyroidism, and rheumatoid arthritis. For the physicians, menopause was central to PMO in that it disrupted bone remodelling, whereas the women spoke of it as one of 11 possible causes of osteoporosis.

When asked about the symptoms of PMO, physicians identified fractures and "weakness" (GP7) as possible symptoms. They nevertheless underlined its "silent," "mute", "asymptomatic" character before the onset of fractures. This feature led some physicians to consider PMO without any fracture to be a "frailness / fragilization" rather than a disease: "*I don’t really consider it a disease*, *in the absence of signs; it’s more a sort of frailness*. *There are no symptoms*. *As long as there is no fracture*, *people don’t actually complain*. *They don’t experience pain*. *There’s no problem with that*.*" (GP3)*.

Moreover, even when a fracture occurred, physicians did not always make the relation between fracture and osteoporosis: orthopedic surgeons were not always concerned about this link and some physicians were uneasy about affirming it. As one stated, a vertebral fracture may be difficult to attribute to osteoporosis: "*You look at cases of vertebral compression*, *but there are so many vertebral diseases now that I’m not sure you can really say that there’s a link to osteoporosis*." (GY5) Even though all of the physicians mentioned fracture, they did not share the same representation of the severity of PMO: some of them stated that not all patients would experience fractures. They also stated that a fracture was not as enduring a condition as other diseases such as rheumatoid arthritis; and they did not consider osteoporosis a fatal disease. Fractures appeared to be the main symptom for the physicians, in contrast to the women who had a broader view, which included pain, curvature of the spine, height loss, loss of mobility, and fatigue.

The physicians considered PMO progression "inevitable" (GP5) and "slow," indicating that there was no urgency in diagnosing or treating it: "*Cancer screenings are among the things that we must remain very*, *very vigilant about*.*…*. *There are things we can’t afford to overlook*, *even if it does happen*. *With osteoporosis*, *we think we have a little time [laughs]; it's a matter of time*.*"(GP3)*. *"There is something insidious about it as it slowly worsens*. *It’s not a cancer that can kill you in 6 months and metastasize*.*"(GP5)*.

When asked about the severity of PMO, practitioners mentioned the occurrence of osteoporotic fractures as a possible starting point for physical degradation. They emphasized: physiological effects (irreparable vertebral compression, irreversible shape change, occurrence of respiratory problems, pain, decreased mobility, and risk of comorbidity); psycho-social effects (decreased sociability, withdrawal, mood changes); and financial implications for society ("a major public health problem"). They recognized the impact of osteoporotic fracture but portrayed PMO as a "benign" disease: "*I know that that's not what's going to kill my patient in any case*." (G1). Compared to other pathologies, osteoporotic fracture and its complications were described as infrequent, limited in time, and generally "repairable": "*It doesn’t have the reputation of being serious; eventually you recover from a fracture*." (R5). Thus, the increased risk of mortality was downplayed, because PMO was perceived as unlikely or "socially acceptable": "*In my view*, *(osteoporosis) is a benign pathology*. . . . *A granny dies of a fracture at 90*, *well*…*she was 90 years old*! *But a 60-year-old woman who dies (of diabetes or a heart condition)*, *she’s lost 30 years of her life*. *Thirty years is a lot*!*"(GP8)*. *"I think the risk of a fracture at 60 is very low*. *That's why I don’t think it's a serious illness*.*" (GP3)*.

Ultimately the interviews showed that the physicians had differing degrees of familiarity with the full range of pharmaceuticals and, like the women, reported fears about anti-osteoporotic treatments. The rheumatologists seemed to be the most likely to master the existing therapeutic arsenal, whereas gynecologists and general practitioners evidenced more limited knowledge. The physicians suggested the following reasons for this: primary care activity, which does not allow for exhaustive knowledge of the therapeutic arsenal for each disease encountered; insufficient exposure to PMO patients; and uncertainty regarding the choice of medication, duration of administration, side effects, and effectiveness: "*Sometimes it's a little difficult to find your way through all of that*. . . . *We don’t feel that the remedies we have available are very effective*." (GP5).

In discussing the compliance issue, the physicians made two different observations. Some stated that they did not encounter problems of treatment compliance, indicating that "*if I tell them to take such-and-such a prescription*, *they take it*." (GY3). They maintained that with the variety of medication available, they could select treatments that corresponded to patients’ expectations in terms of drug burden and obtain compliance. Others, in particular rheumatologists, cited the results of studies carried out on other diseases to convey their impression that breaches of compliance for anti-osteoporotic medications were more common than believed.

The physicians expressed few expectations concerning PMO and its management. The expectations that they did express concerned four areas: training doctors in screening and treating (mentioned by 3 GPs out of 18 physicians); scientific advances in treatment (1 GP): "*Do something acceptable for us regarding treatment*," (GP4); help with therapeutic decision-making and patient education (1 GP): "*Who will take charge of educating patients?*… *You understand*, *it can’t be done as part of a consultation*!" (G4).

## Discussion

Our study identified elements that may impede effective PMO care. In spite of a large number of information campaigns, studies on complications of the disease, and national and international guidelines on indications for treatments, there are still strong barriers to initiating or continuing treatment. We observed that: many still considered osteoporosis as natural bone deterioration, fractures were disconnected to bone fragility, the effects of treatment were not tangible, and patients feared the side effects of medication. Aging rather than disease was perceived as the cause of normal, "natural" deterioration of bones due to wear-and-tear, and this perception was reinforced by the silent, asymptomatic nature of PMO.

The women interviewed showed no particular interest in bones per se, even though they had been diagnosed as having osteoporosis, had had a bone density test, and had been considered in need of treatment. They did not "visualize" their bones. PMO patients might find the disease more discernible if BMD measurements were amply discussed. All the women interviewed indicated that they had been tested but had received little and/or unclear information about the disease. Sale et al. confirmed that the majority of BMD test results were not subsequently discussed with patients or that patients registered unclear or incorrect messages [32]. Significantly, the physicians that we interviewed did not discuss the role of BMD testing in their practice. For BMD testing to be a teaching moment, health providers must be educated concerning: anxiety that BMD test results may cause in subjects with high concern about their health [33]; risk of overtreatment in the most frequent densitometric situation, i.e. osteopenia [34]; and also risk of inappropriate reassuring messages, when the BMD is above the osteoporotic threshold in a patient with a hip or vertebral non-traumatic fracture. BMD measurement is not necessary to treat an elderly frail patient with a non-traumatic hip or vertebral fracture, but telling patients that a BMD test was not necessary was identified as a provider-level barrier to treatment [32, 35].

Our study confirmed the importance of the uncertainty of the relation between fractures and osteoporosis, for the women and for some physicians. The perception that all women with PMO who fell did not sustain fractures could pave the way for a possible reverse interpretation that fractures were not necessarily due to osteoporosis and bone fragility but rather to an impact. Viewing fracture as an outcome of an impact led the women to concentrate on avoiding falling. They perceived fractures as rare, due to impact, risky behaviors, or hazards—an unfortunate event but neither systematically handicapping nor life-threatening. Moreover, some patients questioned taking a treatment for years in order to prevent a rare, random event, which they imagined to be easily treatable. All these elements might explain why experiencing a fracture did not appear to be a segmenting characteristic for PMO representations. Our data agree with the results of a previous qualitative study reporting that patients sustaining fracture often reported explanations linked to falls rather than to bone quality [36]. Sale’s et al. also suggested that the term "fragility fracture" was a misnomer for older adults that impeded patients’ awareness of bone health, and should not be used by health care providers. Physicians therefore face a dilemma: a previous non-traumatic fracture is an indication for anti-osteoporotic treatment, but this recommendation cannot convince a patient who does not link fracture and osteoporosis.

Non-pharmacological strategies such as caution and exercise are used by patients at high risk for future fracture [37]. Our study confirmed women with PMO’s interest in lifestyle changes, which may result in competition between such strategies and anti-osteoporotic treatments. Further studies are needed to assess the role of non pharmacological treatment in adherence to medication.

Negative representations of anti-osteoporotic treatments may have an effect on patient compliance. The side effects of anti-osteoporosis treatments appeared to be a primary source of concern. Although they are very rare, their impact was largely explained by negative publicity in press and media [38], and even a follow-up to rectify a biased presentation did not cancel the effect [25]. A second concern was certain patients’ view that therapeutic breaks indicated the potential danger of a medication. The efficacy of treatment was also a concern for women and for some physicians interviewed: patients wanted some way to gauge the effects of treatment, and some physicians were uncertain about the actual benefits of the medication. These considerations point out the need for more information for patients and training for physicians.

### Strength of the study

Qualitative research has been recommended in order to explore individuals’ beliefs and motivations in medication taking [39], "to provide an in-depth understanding into human behavior" [40], and to reflect the diversity within a given population [41]. It is therefore an appropriate method for providing a deeper understanding of individuals’ views regarding PMO as well as knowledge of 'lay' beliefs about illness. Indeed, our study integrates the main stakeholders’ perspectives, as recommended by Hoang-Kim et al. [42].

Because previous research examining adherence in osteoporosis indicated that patients who agree to take part in research studies are more likely to be adhering to their doctor’s treatment recommendations [43], we paid particular attention to the diversification of our sample. We included women with PMO who refused treatments and who declared breaks in their treatment as well as compliant asymptomatic women in order to select information-rich cases. We also used multiple recruitment strategies to avoid using only medical informants for recruiting in order to enhance diversity in the sample.

### Weaknesses of the study and future research

The results are valid within the French medical care setting. This system is quite favorable to good care of PMO. There is no financial barrier to implementing treatment in that medications are paid for and the bone density test is covered for patients presenting nontraumatic fracture, disease or treatment potentially responsible for osteoporosis, and for postmenopausal women. Comparable research in other cultures and medical systems might, reveal cultural or managerial differences.

A complementary quantification of our results might help appraise the strength of socio-cultural resistances to PMO treatment and identify the socio-demographic profiles that either dramatize or trivialize PMO. A quantitative study would help prioritize areas for improvement in training physicians, in educating and enhancing the awareness of women, and in focusing public communication.

## Conclusion

This study suggests several potential improvements in patient management and physician training. A better understanding of the women’s views and practices related to PMO can help physicians work in an ongoing partnership with their patients, addressing their actual concerns and expectations. More attention and time should be devoted to patients’ concerns and representations in order to better understand their priorities [44], including their fears concerning treatment. The findings may be useful for improving therapeutic education for women with PMO. They could also be used to tailor targeted information, enabling physicians to improve their patients’ awareness of fracture outcomes and medication breaks and to better optimize drug therapies.

## Supporting Information

S1 Table Women’s characteristics. (DOCX)

S2 Table Declared anti-osteoporotic medications. (DOC)
