# Supplementary material for: Barriers to Effective Postmenopausal Osteoporosis Treatment: A Qualitative Study of Patients’ and Practitioners’ Views
Source: PLoS One. 2016 Jun 29;11(6):e0158365. doi: 10.1371/journal.pone.0158365 (PMC4927112; doi:10.1371/journal.pone.0158365)
Supplement: S1 Table — (DOCX) [file pone.0158365.s001.docx]

S1. Women’s characteristics (at interview date)

| **Code** | **Age** | **Occupation** | **Current work status** | **Family status** | **Data collection method** | **Living area** | **Declared fractures** | **Declared treatment compliance** |
| --- | --- | --- | --- | --- | --- | --- | --- | --- |
| W1 | 74 | Housewife | Retired | Married | SSI* | Semi-rural | — | Compliant |
| W2 | 67 | Journalist | Retired | Divorced | SSI | Urban | Wrist | Compliant |
| W3 | 79 | — | Retired | Married | SSI | Semi-rural | — | Non-compliant |
| W4 | 71 | Medical secretary | Retired | Married | SSI | Urban | — | Compliant |
| W5 | 74 | Trainer | Retired | Divorced | SSI | Urban | Wrist, elbow, rib | Compliant |
| W6 | 70 | Technical illustrator | Retired | Single | SSI | Urban | — | Non-compliant |
| W7 | 63 | Secretary | Retired | Divorced | SSI | Urban | — | Non-compliant |
| W8 | 66 | Real estate agent | Retired | Divorced | SSI | Urban | — | Non-compliant |
| W9 | 76 | Housewife | Non working | Widow | SSI | Urban | Malleolus, humerus, elbow | Compliant |
| W10 | 64 | Pension fund employee | Retired | Single | SSI | Urban | — | Non-compliant |
| W11 | 64 | Secretary | Retired | Single | SSI | Urban | Hip, foot | Non-compliant |
| W12 | 68 | Secretary | Retired | Married | SSI | Urban | Ankle, foot | Non-compliant |
| W13 | 76 | Shop owner | Retired | Married | SSI | Urban | Hip, elbow | Non-compliant |
| W14 | 66 | Real estate agent | Retired | Married | SSI | Urban | Vertebra | Non-compliant |
| W15 | 75 | Secretary | Retired | Widow | SSI | Urban | Wrist, kneecap | Non-compliant |
| W16 | 61 | Insurance management officer | Retired | Divorced | SSI | Urban | Toes, coccyx | Compliant |
| W17 | 76 | Saleswoman (dental sector) | Retired | Divorced | SSI | Urban | Vertebrae | Non-compliant |
| W18 | 79 | Private insurance employee | Retired | Widow | SSI | Rural | — | Non-compliant |
| W19 | 63 | Art historian and free-lance interpreter | Irregular, freelance work | Married | SSI | Urban | — | Compliant |
| W20 | 72 | Bank employee | Retired | Widow | SSI | Semi-rural | — | Non-compliant |
| W21 | 63 | School teacher | Retired | Married | SSI | Semi-rural | — | Compliant |
| W22 | 62 | Private nurse | Working | Divorced | SSI | Urban | — | Non-compliant |
| W23 | 78 | Accountant | Retired | Married | SSI | Semi-rural | — | Compliant |
| W24 | 79 | Gallerist | Retired | Widow | SSI | Urban | — | Non-compliant |
| W25 | 87 | Shopkeeper | Retired | Married | SSI | Urban | Hip | Compliant |
| W26 | 56 | Sales assistant | Working | Married | SSI | Rural | Hip | Compliant |
| W27 | 55 | Service officer | Working | Married | SSI | Urban | Hip, vertebra | Non-compliant |
| W28 | 75 | Farmer | Retired | Married | SSI | Rural | Hip | Compliant |
| W29 | 76 | Interpreter / translator | Retired | Widow | SSI | Rural | Hip, humerus, vertebrae, ankle | Compliant |
| W30 | 56 | Executive secretary | Working | Married | FG** | Urban | — | Compliant |
| W31 | 61 | Executive assistant | Retired | Cohabiting | FG | Urban | — | Compliant |
| W32 | 58 | Executive assistant | Working | Married | FG | Urban | — | Compliant |
| W33 | 60 | Executive assistant | Retired | Married | FG | Urban | — | Compliant |
| W34 | 60 | Housewife | Nonworking | Married | SSI | Urban | — | Compliant |
| W35 | 61 | Civil servant / records manager | Retired | Married | SSI | Semi-rural | — | Non-compliant |
| W36 | 63 | School teacher | Retired | Divorced | SSI | Urban | — | Compliant |
| W37 | 60 | Nursery assistant | Nonworking on sick leave | Married | SSI | Urban | Clavicle, rib | Non-compliant |

* SSI: semi-structured interview

**FG: focus group
