# Supplementary material for: Barriers to Effective Postmenopausal Osteoporosis Treatment: A Qualitative Study of Patients’ and Practitioners’ Views
Source: PLoS One. 2016 Jun 29;11(6):e0158365. doi: 10.1371/journal.pone.0158365 (PMC4927112; doi:10.1371/journal.pone.0158365)
Supplement: S2 Table — (DOC) [file pone.0158365.s002.doc]

S2. Declared anti-osteoporotic medications (at interview date)

| **Code** | **1^st^ treatment** | **1st treatment duration** | **2^nd^ treatment** | **2^nd^ treatment duration** | **3^rd^ treatment** | **3^rd^ treatment duration** | **4^th^ treatment** | **4^th^ treatment duration** | **5^th^ treatment** | **5^th^ treatment duration** | **6^th^ treatment** | **6^th^ treatment duration** | **7^th^ treatment** | **7^th^ treatment duration** |
| --- | --- | --- | --- | --- | --- | --- | --- | --- | --- | --- | --- | --- | --- | --- |
| W1 | strontium ranelate | "3 yrs" |  |  |  |  |  |  |  |  |  |  |  |  |
| W2 | risedronate | "7-8 yrs" | denosumab | "a few months" |  |  |  |  |  |  |  |  |  |  |
| W3 | alendronate | "some time" | strontium ranelate | "2 yrs" | denosumab proposed | refused |  |  |  |  |  |  |  |  |
| W4 | risedronate | "3 yrs" |  |  |  |  |  |  |  |  |  |  |  |  |
| W5 | THS | "7 yrs" | risedronate | "7 yrs" | strontium ranelate | "6 months" | risedronate | "9 yrs" | alendronate | "9 yrs" | denosumab | "1 yr" | zoledronic acid | "1 yr" then therapeutic break |
| W6 | risedronate | "2 yrs" | alendronate | "5 yrs" |  |  |  |  |  |  |  |  |  |  |
| P7 | THS | "several years" | alendronate | "7 to 8 yrs" |  |  |  |  |  |  |  |  |  |  |
| W8 | alendronate | "2 yrs" | alendronate | "1 yr" |  |  |  |  |  |  |  |  |  |  |
| W9 | alendronate | "3 yrs" | risedronate | "10 yrs" | alendronate | "3 yrs" | strontium ranelate | "3 yrs" | denosumab | "a few months" |  |  |  |  |
| W10 | ibandronate | "6-7 yrs" |  |  |  |  |  |  |  |  |  |  |  |  |
| W11 | strontium ranelate | doesn’t know | risedronate | doesn’t know | alendronate | doesn’t know |  |  |  |  |  |  |  |  |
| W12 | ibandronate | "at least 10 yrs" |  |  |  |  |  |  |  |  |  |  |  |  |
| W13 | alendronate | "1 week" |  |  |  |  |  |  |  |  |  |  |  |  |
| W14 | THS | "several years" | strontium ranelate | "2-3 yrs" | alendronate | "2 or 3 yrs" | raloxifène, denosumab, & risedronate proposed | refused |  |  |  |  |  |  |
| W15 | THS | unspecified but not taken on a regular basis | alendronate | "more than 10 yrs" | ibandronate | "1 yr" | strontium ranelate proposed | refused |  |  |  |  |  |  |
| W16 | raloxifène | "10 yrs" | risedronate | "a few months" | raloxifène |  |  |  |  |  |  |  |  |  |
| W17 | alendronate | "7 weeks" | risedronate | "2 weeks" | denosumab proposed | refused |  |  |  |  |  |  |  |  |
| W18 | THS | "15 yrs" | strontium ranelate | doesn’t know |  |  |  |  |  |  |  |  |  |  |
| W19 | risedronate | "5 yrs" | therapeutic break | "1 or 2 yrs" | zoledronic acid | "1 yr" | risedronate | "1 yr" |  |  |  |  |  |  |
| W20 | raloxifène | "2 months" | alendronate | "2 months" | zoledronic acid proposed | refused |  |  |  |  |  |  |  |  |
| W21 | raloxifène | "3 yrs" |  |  |  |  |  |  |  |  |  |  |  |  |
| W22 | THS proposed | refused | zoledronic acid proposed | refused |  |  |  |  |  |  |  |  |  |  |
| W23 | THS | "10 yrs" | ibandronate | "6 yrs" | alendronate | "3 yrs" |  |  |  |  |  |  |  |  |
| W24 | THS | "10 yrs" | strontium ranelate | "8 days" | Zoledronic acid & denosumab proposed | refused |  |  |  |  |  |  |  |  |
| W25 | teriparatide | "for quite some time" |  |  |  |  |  |  |  |  |  |  |  |  |
| W26 | risedronate | "a few months" |  |  |  |  |  |  |  |  |  |  |  |  |
| W27 | strontium ranelate | "1 yr" | bisphosphonate proposed | refused |  |  |  |  |  |  |  |  |  |  |
| W28 | THS | "10 yrs" | zoledronic acid | "2 yrs" |  |  |  |  |  |  |  |  |  |  |
| W29 | THS | unspecified (still taking) | risedronate | unspecified | teriparatide | "2 yrs" | zoledronic acid | "a few months" |  |  |  |  |  |  |
| W30 | risedronate | "4 yrs" | alendronate | "1.5 yr" |  |  |  |  |  |  |  |  |  |  |
| W31 | ibandronate | unspecified |  |  |  |  |  |  |  |  |  |  |  |  |
| W32 | raloxifène | unspecified |  |  |  |  |  |  |  |  |  |  |  |  |
| W33 | risedronate | "2 yrs" |  |  |  |  |  |  |  |  |  |  |  |  |
| W34 | zoledronic acid | "5 yrs" | therapeutic break | ongoing |  |  |  |  |  |  |  |  |  |  |
| W35 | THS | "8 yrs" | “break for 1 yr" | ongoing |  |  |  |  |  |  |  |  |  |  |
| W36 | alendronate | "6 yrs" | therapeutic break | ongoing |  |  |  |  |  |  |  |  |  |  |
| W37 | zoledronic acid proposed | refused | risedronate proposed | refused |  |  |  |  |  |  |  |  |  |  |
